# Recombinant Immunogens Designed by AI Epitope Prioritization Confer Protection Against *Mycobacterium tuberculosis*

**DOI:** 10.3390/vaccines14050408

**Published:** 2026-05-01

**Authors:** Ning Fang, Anke Chen, Zhifei Zhang, Menglin Ye, Jiasong Pan, Weili Huang, Decheng Wang, Zhidong Hu, Xiaoyong Fan, Bingdong Zhu, Ying Wang, Guoping Zhao, Lu Zhang, Jixi Li

**Affiliations:** 1 Shanghai Pudong Hospital and School of Life Sciences, State Key Laboratory of Genetics and Development of Complex Phenotypes, Shanghai Engineering Research Center of Industrial Microorganisms, Fudan University, Shanghai 200438, China; 24110700013@m.fudan.edu.cn (N.F.); 22110700003@m.fudan.edu.cn (A.C.); zhangzhifei@fudan.edu.cn (Z.Z.); mlye23@m.fudan.edu.cn (M.Y.); 19210700095@fudan.edu.cn (J.P.); 2 School of Life Sciences, State Key Laboratory of Genetics and Development of Complex Phenotypes, Shanghai Engineering Research Center of Industrial Microorganisms, Fudan University, Shanghai 200438, China; weilihuang98@163.com (W.H.); gpzhao@sibs.ac.cn (G.Z.); 3 College of Basic Medical Sciences, China Three Gorges University, Yichang 443002, China; dcwang@fudan.edu.cn; 4 Shanghai Institute of Infectious Diseases and Biosecurity, Shanghai Public Health Clinical Center, Fudan University, Shanghai 201508, China; huzhidong@fudan.edu.cn (Z.H.); xyfan008@fudan.edu.cn (X.F.); 5 State Key Laboratory for Animal Disease Control and Prevention, Lanzhou Center for Tuberculosis Research, Institute of Pathogen Biology, School of Basic Medical Sciences, Lanzhou University, Lanzhou 730000, China; bdzhu@lzu.edu.cn; 6 Shanghai Institute of Immunology, Shanghai Jiao Tong University, Shanghai 200025, China; ywang@sibs.ac.cn

**Keywords:** tuberculosis, AI-guided, recombinant immunogens, RI-13 vaccine

## Abstract

**Background:** Tuberculosis (TB) remains a major global health challenge, highlighting the urgent need for more effective vaccines. This study aimed to develop an artificial intelligence-guided epitope prediction and prioritization pipeline to identify immunodominant peptides from *Mycobacterium tuberculosis* (Mtb) and to evaluate the immunogenicity and protective efficacy of the resulting vaccine candidates. **Methods:** An AI-guided framework was used to predict and prioritize immunodominant Mtb epitopes, leading to the generation of 72 recombinant immunogens. Among these, RI-13, RI-20, and RI-31 were selected as the leading candidates. Their protective efficacy was assessed in a zebrafish TB infection model and in BALB/c mice following DNA vaccination. Humoral and cellular immune responses were further evaluated in C57BL/6 mice. **Results:** RI-13, RI-20, and RI-31 markedly reduced infection-associated pathology and lowered bacterial burden by up to 1.5 log_10_ in the zebrafish TB infection model, outperforming benchmark antigen combinations, including the Ag85A plus ESAT6/CFP10 cocktail used in the phase III vaccine candidate GamTBvac. In BALB/c mice, DNA vaccination with each construct reduced pulmonary mycobacterial burden by approximately 0.3 log_10_ and alleviated lung tissue damage. In addition, all three candidates elicited robust humoral and cellular immune responses, with RI-13 showing the strongest overall immunogenicity and inducing a balanced Th1, Th2, and Th17 response profile in C57BL/6 mice. **Conclusions:** These findings identify RI-13, derived from Rv1174c, as a promising next-generation TB vaccine candidate. More broadly, this study supports the utility of an AI-guided framework for the rational design and preclinical prioritization of novel TB immunogens.

## 1. Importance

Tuberculosis remains a leading infectious killer, and the only licensed vaccine, BCG, provides inconsistent protection against adult pulmonary disease. New TB vaccines are urgently needed, but identifying antigens that induce protective immunity remains a major bottleneck. Here, we used an artificial intelligence-guided epitope discovery pipeline to identify *Mycobacterium tuberculosis* peptides and engineer three recombinant immunogens with in vivo protective activity. These candidates reduced disease-associated pathology in zebrafish and lowered mycobacterial burden and lung damage in mice. RI-13 emerged as the strongest lead, eliciting robust humoral and cellular immunity with a balanced Th1, Th2, and Th17 profile. Beyond identifying a promising next-generation TB vaccine candidate, this study establishes a scalable framework for rational antigen selection and rapid preclinical prioritization. Our findings show how AI-guided immunogen design can accelerate TB vaccine development and may be broadly useful for other difficult infectious diseases.

## 2. Introduction

Tuberculosis (TB), caused by *Mycobacterium tuberculosis* (Mtb), remains a leading cause of infectious disease mortality worldwide [1,2,3]. While pulmonary TB (PTB) is the predominant clinical manifestation, systemic dissemination and chronic progression make TB a major global health burden, responsible for nearly twice as many deaths as HIV/AIDS [4]. Despite decades of control efforts, approximately one-quarter of the global population remains latently infected with Mtb [4,5].

Current chemotherapeutic regimens, based on first-line agents such as rifampicin, isoniazid, pyrazinamide, and ethambutol, have significantly reduced disease burden [6,7]. However, the rise in multidrug-resistant (MDR) and extensively drug-resistant (XDR) Mtb strains has eroded the efficacy of these treatments and exposed vulnerabilities in TB control strategies [8,9]. As drug resistance spreads and treatment compliance remains challenging, preventative vaccination is increasingly recognized as the most effective long-term strategy for TB eradication [10,11,12].

The Bacille Calmette-Guérin (BCG) vaccine, a live attenuated *Mycobacterium bovis* strain, remains the only licensed TB vaccine in use [13]. However, its protective efficacy is highly variable (0–80%) and largely fails to protect adolescents and adults against pulmonary TB, the primary driver of Mtb transmission [14]. This has spurred the global development of next-generation TB vaccines, including inactivated, live-attenuated, recombinant BCG, subunit, viral vector, and nucleic acid-based forms [11,15,16,17,18,19]. Among these, subunit vaccines show considerable promise due to their defined composition, improved safety profiles, and compatibility with rational design.

Recent advances in computational immunology and artificial intelligence (AI)-guided epitope prediction have enabled systematic discovery of antigenic targets from Mtb and other pathogens [20,21]. However, the resulting immune efficacy remains limited due to insufficient induction of effective humoral responses. In this study, we employed an AI-assisted pipeline to identify dominant B-cell and T-cell epitopes from Mtb proteins and engineered three recombinant immunogens, RI-13, RI-20, and RI-31. These immunogens were validated in zebrafish and murine TB models for their ability to elicit protective immune responses. Our findings demonstrate the feasibility of an AI-driven design pipeline for TB subunit vaccine development and identify RI-13 as a promising candidate for further preclinical advancement.

## 3. Materials and Methods

### 3.1. Zebrafish and Mice

Wild-type adult zebrafish (*Danio rerio*, AB strain, 3–4 months old) were obtained from Nanjing Yishulihua Biotechnology Co., Ltd. (Nanjing, China). All zebrafish were maintained under standard laboratory conditions prior to experimentation. Female BALB/c and C57BL/6 mice (6–8 weeks old) were purchased from SPF Biotechnology Co., Ltd. (Beijing, China). Mice were housed under specific pathogen-free (SPF) conditions and acclimatized in the animal facility for at least one week before the initiation of experiments.

### 3.2. Bacteria and Cell Lines

*M. marinum* 535, *M. bovis* BCG, and *M. tuberculosis* H37Rv (Shanghai Key Laboratory of Tuberculosis, China) were cultured in Middlebrook 7H9 broth (BD, Franklin Lakes, NJ, USA) supplemented with 0.2% glycerol and 10% OADC enrichment (oleic acid, bovine serum albumin, dextrose, and catalase; Difco, Sparks, MD, USA). HEK293T cells were maintained in Dulbecco’s Modified Eagle Medium (DMEM) supplemented with 10% fetal bovine serum (FBS) and 1% penicillin-streptomycin. HEK293F cells were cultured in 293F Hi-Exp medium containing 1% FBS under suspension conditions.

### 3.3. Prediction of B-Cell and T-Cell Epitopes for Tuberculosis Antigen

B-cell epitopes were predicted using the enhanced SEPPA 3.0 platform (http://www.badd-cao.net/seppa3/, accessed on 30 April 2026) [22], which incorporates structural features and glycosylation-site information. For antigens with experimentally resolved structures available in the Protein Data Bank (PDB), the corresponding PDB entries were directly submitted to SEPPA 3.0. For antigens lacking experimental structural data, high-confidence structural models were generated using AlphaFold3 (https://alphafoldserver.com, accessed on 30 April 2026) [23] These predicted structures were carefully evaluated, and low-confidence regions were trimmed or adjusted before submission to SEPPA 3.0. Human was selected as the immune host, and the output provided a set of putative B-cell epitopes for each antigen.

T-cell epitopes were predicted using the T Cell Epitope Prediction tools available through the Immune Epitope Database (IEDB). Amino acid sequences of *Mycobacterium tuberculosis* antigens were submitted for prediction of both MHC class I- and MHC class II-restricted epitopes, with human selected as the host species. The output consisted of ranked epitope candidates with associated binding scores or percentile ranks.

Approximately 4000 annotated *M. tuberculosis* proteins were used as the initial antigen pool. For each antigen sequence, in silico prediction of MHC-I-restricted T-cell epitopes, MHC-II-restricted T-cell epitopes, and linear B-cell epitopes was performed. For MHC-I epitope prediction, the source species was set to human, and all available HLA alleles were included. Predicted peptides were ranked by score, and the top three peptides with the highest scores were selected. For MHC-II epitope prediction, peptide lengths of 12–18 amino acids were evaluated. Predicted peptides were ranked by percentile rank, and the top three peptides with the lowest percentile ranks were selected. For B-cell epitope prediction, the top three continuous peptide segments with the highest scores were selected.

The selected three MHC-I peptides, three MHC-II peptides, and three B-cell peptide segments were then merged to generate a new full-coverage chimeric antigen sequence for each protein. These redesigned sequences were used as candidate immunogens and were subsequently combined with ESAT-6/CFP-10 for downstream construct generation and experimental testing.

### 3.4. Construction of Recombinant Immunogens

A total of 72 recombinant immunogens were computationally designed by concatenating predicted B-cell and T-cell epitope peptides. Each construct was fused at the N-terminus to *M. tuberculosis* ESAT-6 and CFP-10 antigens via a flexible linker (GSGGSGSGGS). A C-terminal 6×His tag was included to facilitate purification. All recombinant immunogen sequences were synthesized by Tsingke (Beijing, China) and cloned into the mammalian expression vector pcDNA3.1 for downstream applications.

### 3.5. DNA Vaccine Immunization in Zebrafish

Adult AB strain zebrafish (3–4 months old) were immunized twice at two-week intervals (weeks 0 and 2). Each fish received a 2 μL intramuscular injection containing 6 μg of DNA vaccine (purified DNA without adjuvants or other additives) into the dorsal muscle using a PV830 Pneumatic PicoPump microinjector (World Precision Instruments, Sarasota, FL, USA). Immediately following injection, the target tissue was electroporated using six pulses of 50 V for 5 ms each, delivered via tweezer-type electrodes (BTX/Harvard Apparatus) connected to a GenePulser electroporator (Bio-Rad, Hercules, CA, USA). For controls, zebrafish injected with empty pcDNA3.1 plasmid served as the negative control group, while those injected with pcDNA-E6C10 plasmid served as the positive control group.

### 3.6. M. marinum Infections in Zebrafish

*M. marinum* infections were conducted two weeks after the final immunization. Zebrafish were fasted for 24 h prior to infection and resumed feeding one day post-infection. For infection, zebrafish were anesthetized in tricaine solution (Sigma, St. Louis, MO, USA) for 30 s and positioned on a microinjection stage. Using a microneedle, 2 μL of *M. marinum* suspension (approximately 800 CFU per fish) was injected into the abdominal cavity, with the injection site located just above the cloaca. The depth of needle insertion was carefully controlled between 0.5 and 0.8 cm to ensure consistent delivery. After injection, zebrafish were transferred to a recovery tank and monitored for survival for up to 60 days post-infection.

### 3.7. DNA Vaccine Immunization in Mice

BALB/c mice were randomly assigned to five groups (n = 6 per group) and housed under specific pathogen-free (SPF) conditions for at least one week to allow for acclimatization. Mice received three intramuscular injections of the DNA vaccine in the left hind limb at weeks 0, 2, and 4. Each immunization consisted of 100 μL of DNA solution containing 100 μg of plasmid DNA without adjuvants or other additives.

### 3.8. Mtb H37Rv Infections in Mice

Four weeks after the final DNA immunization, BALB/c mice were subjected to aerosol challenge with *M. tuberculosis* H37Rv. Mice were placed in an aerosol exposure chamber and exposed to aerosolized Mtb H37Rv for 10 min. Following exposure, the system was turned off and allowed to settle for 5 min to ensure complete aerosol deposition. Each mouse received an estimated inhaled dose of approximately 200 colony-forming units (CFU) of Mtb H37Rv.

Four weeks post-infection, mice were euthanized using CO_2_ asphyxiation with cervical dislocation for confirmation. Next, complete necropsy was performed to aseptically harvest lungs and spleens. The organs were rinsed with sterile PBS and individually homogenized. The resulting homogenates were serially diluted and plated onto 7H10 agar medium. Plates were incubated inverted at 37 °C for 4 weeks before enumeration of bacterial colonies to determine organ-specific mycobacterial loads.

### 3.9. Histopathological Analysis

Zebrafish were euthanized in tricaine solution. Specimens were rinsed once with sterile PBS and fixed in 4% paraformaldehyde at 4 °C for 1 week. After fixation, samples were washed under running water for 12 h and decalcified in 10% ethylenediaminetetraacetic acid (EDTA) for 1 week. Samples were then dehydrated through a graded ethanol series and cleared through a graded xylene series. For paraffin infiltration, specimens were incubated in molten paraffin at 60 °C for 2 h, transferred to fresh paraffin, and incubated for an additional 12 h to remove residual xylene. Samples were embedded in molds filled with molten paraffin. After blocks solidified at room temperature, they were chilled at −30 °C for 5 min, demolded, and sent to Wuhan Servicebio Technology Co., Ltd. (Wuhan, China). for sagittal sectioning.

Hematoxylin and eosin (H&E) staining was performed as follows. Sections were deparaffinized and stained with hematoxylin for 5 min, then rinsed under running water for bluing. Sections were briefly differentiated in 1% hydrochloric acid in alcohol for 2 s and rinsed again under running water until the background cleared. Sections were then stained with eosin for 5 s and washed thoroughly. After air drying, sections were cleared sequentially in Van-Clear I (Wuhan Servicebio Technology Co., Ltd., Wuhan, China) for 10 min and Van-Clear II for 5 min, mounted with mounting medium, and examined by light microscopy.

For acid-fast staining, sections containing intact visceral structures were selected. After deparaffinization, sections were incubated in carbol fuchsin for 20 min and rinsed under running water. Sections were differentiated in acid alcohol for 5 s, rinsed, counterstained with methylene blue for 5 s, and rinsed again. Sections were air-dried, mounted with mounting medium, and examined by microscopy.

### 3.10. Subunit Vaccine Immunization in Mice

The target proteins (RI-13, RI-20, and RI-31) were diluted to a final concentration of 0.2 mg/mL in PBS. For each immunization dose, 600 μL of the diluted protein solution was mixed with 600 μL of Freund’s adjuvant. Complete Freund’s adjuvant was used for the primary immunization, and incomplete Freund’s adjuvant was used for the subsequent booster immunizations. The mixture was kept on ice and emulsified using a handheld ultrasonic homogenizer, with each pulse lasting no longer than 10 s to prevent overheating. Emulsification was completed within approximately 10 min. C57BL/6 mice received three intramuscular injections of the subunit vaccine into the left hind limb, administered at weeks 0, 2, and 4. Each injection consisted of 100 μL of the emulsified vaccine formulation.

### 3.11. ELISA Antibody Titer Detection

Antibody titers in mouse serum were determined using an ELISA assay. Recombinant immunogen proteins were diluted to 1 ng/μL in 1× ELISA coating buffer. After appropriate dilution with water, 100 μL of the mixture was added to each well of a 96-well plate and incubated at 4 °C for at least 16 h for coating. Following incubation, the wells were washed three times with PBST (PBS + 0.05% Tween-20), and then 200 μL of blocking solution was added to each well and incubated at 37 °C for 1 h.

After blocking, wells were washed and 100 μL of serially diluted mouse serum samples (dilutions ranging from 1:100 to 1:12,800 in sample diluent) were added to each well and incubated at 37 °C for 2 h. The wells were then washed three times with PBST. Subsequently, 100 μL of goat anti-mouse IgG-HRP secondary antibody (diluted 1:10,000 in sample diluent) was added and incubated at 37 °C for 1 h. After washing, 100 μL of TMB substrate was added to each well, and the reaction was allowed to proceed in the dark for no more than 15 min. The reaction was stopped by adding 100 μL of stop solution, and absorbance was measured at 450 nm using an ELISA plate reader.

### 3.12. Cytokine Detection with ELISA

Cytokine levels in splenic lymphocyte culture supernatants were measured by ELISA. Briefly, 4 × 10^5^ isolated splenic lymphocytes were seeded into each well of a 24-well plate in a final volume of 300 μL of culture medium. For antigen stimulation, 10 μL of 0.3 mg/mL protein was added to the designated wells, thoroughly mixed, and incubated at 37 °C for 48 h. Supernatants were collected by centrifugation and used for ELISA analysis.

For ELISA, 100 μL of each cell culture supernatant was added to a 96-well plate, with one column reserved for a serial dilution of cytokine standards. Plates were incubated at 37 °C for 90 min. After removing the liquid, 100 μL of cytokine-specific detection antibody was added to each well and incubated at 37 °C for 60 min. Wells were then washed three times with 200 μL of 0.01 M TBS. Subsequently, 100 μL of Avidin-Biotin Complex (ABC) reagent was added and incubated at 37 °C for 30 min, followed by five washes with 0.01 M TBS. For signal development, 100 μL of TMB substrate solution was added to each well and allowed to react in the dark for up to 30 min. The reaction was stopped by adding 100 μL of TMB stop solution, and absorbance was measured at 450 nm using a microplate reader (Molecular Devices Instrument Co., San Jose, CA, USA).

### 3.13. Flow Cytometry Analysis of Mouse Splenic Lymphocytes

Flow cytometry was performed to analyze mouse splenic lymphocytes. Briefly, 2 × 10^6^ splenic lymphocytes were seeded into each well of a 24-well plate with 500 μL of culture medium. For antigen stimulation, 10 μL of 0.5 mg/mL protein was added to designated wells. Separate wells were set aside for positive controls, single-stain controls, and fluorescence minus one (FMO) controls. After 20 h of incubation, 5 μL of Monensin Solution (100× in DMSO) was added per well and incubated for an additional 6 h. Positive control wells received 5 μL of 5 μg/mL PMA/PKA and 5 μL of 50 μg/mL ionomycin simultaneously with Monensin.

After stimulation, both cells and supernatants were collected by centrifugation at 1500 rpm for 5 min at 4 °C, and supernatants were discarded. Cells were washed once with 200 μL PBS, resuspended in 100 μL PBS, and blocked with 1 μL TruStain FcX™ (anti-mouse CD16/32, BioLegend, San Diego, CA, USA) for 15 min at 4 °C in the dark. After another wash, cells were stained with 1 μL Zombie Aqua™ (BioLegend, San Diego, CA, USA) viability dye for 15 min under the same conditions. Next, 100 μL of surface antibody staining mix was added to each well and incubated for 30 min at 4 °C in the dark. Cells were washed with 200 μL Staining Buffer and fixed in 500 μL Fixation Buffer for 20 min at room temperature in the dark. After fixation, cells were washed and permeabilized twice using 1× Intracellular Staining Perm Wash Buffer.

Intracellular staining was performed by adding 100 μL of cytokine antibody mix and incubating for 30 min at 4 °C in the dark. Cells were washed once with Perm Wash Buffer and once with Staining Buffer, then resuspended in 300 μL Staining Buffer, filtered through a mesh, and analyzed by flow cytometry. Gating and voltage settings were optimized using single-stain and FMO controls, and all samples were analyzed sequentially.

### 3.14. Protein Expression and Purification

HEK293F-expressed proteins: E6C10, RI-13, RI-20, and RI-31 were expressed in HEK293F cells. Cells were seeded at a density of 2 × 10^6^ cells/mL and transfected using the Lipo293F™ transfection reagent (Beyotime, Shanghai, China), following the manufacturer’s protocol. After 72 h, cells were harvested by centrifugation at 3000 rpm for 15 min at 4 °C. Pellets were resuspended in lysis buffer (1× PBS, 10 mM imidazole, pH 8.0) and lysed using a high-pressure homogenizer (JNBIO, Jinan, China). Cell debris was removed by centrifugation at 17,000 rpm for 60 min at 4 °C. The clarified lysate was applied to a HisTrap™ HP column (GE Healthcare, Chicago, IL, USA), and the target proteins were eluted using buffer containing 250 mM imidazole (1× PBS, pH 8.0). Eluted proteins were further purified by size-exclusion chromatography using a Superdex 200 10/300 column (GE Healthcare) in buffer (20 mM Tris-HCl, 100 mM NaCl, pH 8.0).

### 3.15. Western Blotting

HEK293T cells were transfected with E6C10, RI-13, RI-20, or RI-31 plasmids. After 24 h, the culture medium was removed, and cells were washed with PBS and collected into 1.5 mL Eppendorf tubes. Cells were lysed in 150 μL RIPA buffer (Beyotime) supplemented with a protease inhibitor cocktail (Beyotime) at 4 °C on a shaker for 30 min. Lysates were centrifuged at 12,000× *g* for 30 min at 4 °C, and supernatants were collected. Protein samples were mixed with 5× loading buffer, boiled at 95 °C for 10 min, and subjected to SDS-PAGE. Proteins were transferred and analyzed by Western blot using the following antibodies: mouse anti-His (1:5000, Proteintech, Wuhan, China), rabbit anti-ACTIN (1:5000, Proteintech), and HRP-conjugated goat anti-mouse IgG(H+L) secondary antibody (1:10,000, Proteintech).

## 4. Results

### 4.1. Strategies for Constructing Anti-Tuberculosis Recombinant Immunogens

**Figure 1:**
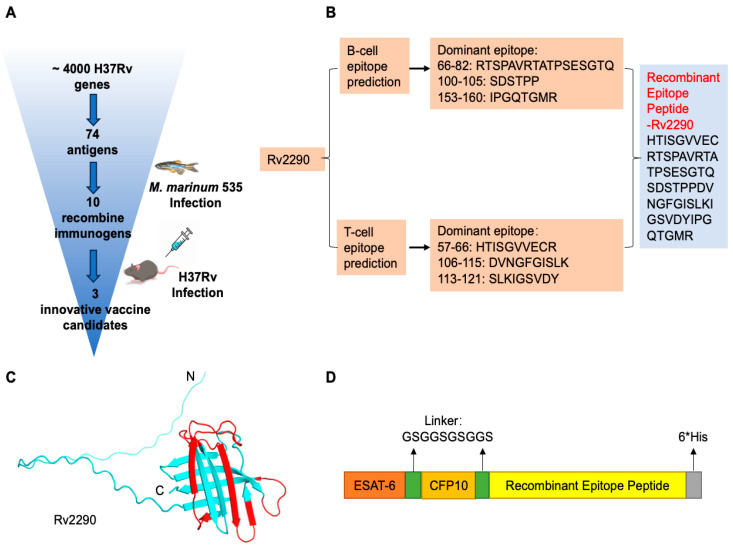
Strategy for Constructing Anti-Tuberculosis Recombinant Immunogens. (**A**) Schematic workflow of antigen screening and selection. (**B**) Epitope prediction of Rv2290 using the IEDB (T-cell) and SEPPA 3.0 (B-cell), followed by rational recombination of predicted epitopes for vaccine design. (**C**) Structural modeling of Rv2290 by AlphaFold3, with predicted epitope regions highlighted in red. (**D**) Design of the multi-epitope recombinant immunogen sequence incorporating ESAT-6, CFP-10, and Rv2290 epitopes connected by flexible linkers.

Protective immunity against *M. tuberculosis* (Mtb) requires coordinated cellular and humoral responses. To rationally design subunit vaccine candidates that can elicit both arms of the adaptive immune system, we established a multi-step epitope-driven immunogen design pipeline incorporating antigen prioritization, computational epitope prediction, and recombinant construct engineering. Using the SEPPA 3.0 platform and the IEDB, we systematically predicted the B-cell and MHC class I/II T-cell epitopes across an unbiased comprehensive database of ~4000 annotated Mtb antigens. A panel of 74 Mtb proteins was enriched in predicted human T-cell and B-Cell epitopes by combining the threshold values (Figure 1A, Table S1). This set included canonical immunodominant antigens Rv3874 (ESAT-6) and Rv3875 (CFP-10), the major components of the phase III vaccine candidate GamTBvac [24,25], as well as 72 additional proteins with high predicted immunogenicity.

Taking Rv2290 as a representative example, we employed AlphaFold3 to model its 3D structure and used SEPPA 3.0 to identify dominant conformational B-cell epitopes (Figure 1B,C). The top-ranking linear B-cell epitopes were localized to amino acid regions 66–82, 100–105, and 153–160 (RTSPAVRTATPSESGTQ, SDSTPP, and IPGQTGMR, respectively). T-cell epitope prediction via IEDB yielded three high-affinity MHC-binding peptides: 57–66 (HTISGVVECR), 106–115 (DVNGFGISLK), and 113–121 (SLKIGSVDY) (Figure 1B). We recombined the top B-cell and T-cell epitopes into a contiguous synthetic peptide, eliminating non-epitopic regions. To generate multivalent constructs, this recombinant Rv2290 peptide was fused with ESAT-6 and CFP-10 using flexible GSGGSGSGGS linkers, and a C-terminal 6×His tag was appended for purification in the eukaryotic expression vector pcDNA3.1 (Figure 1D) [26]. This workflow was systematically applied to all 72 selected antigens. Expression constructs were transfected into HEK293T cells and screened for stable protein expression by Western blotting. Among the 72 designed immunogens, 20 candidates with negligible or low expression were excluded according to their expression levels and Western blotting results. 52 demonstrated robust expression in mammalian cells and were designated as recombinant immunogens RI-1 through RI-52 (Figure S1, Table S2). These candidates were selected for further functional and immunological evaluation in vivo.

### 4.2. RI-13, RI-20, and RI-31 Confer Protection Against M. marinum Infection in Zebrafish

**Figure 2:**
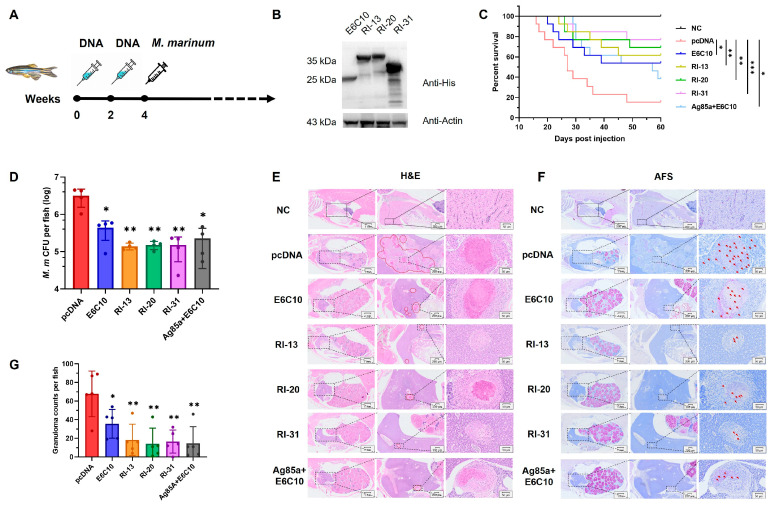
RI-13, RI-20, and RI-31 Confer Protection Against *M. marinum* Infection in Zebrafish. (**A**) Immunization and infection timeline in zebrafish. (**B**) Western blot showing expression of E6C10, RI-13, RI-20, and RI-31 in HEK293T cells at 48 h post-transfection. (**C**) Survival curves of adult zebrafish (n = 15) immunized twice intramuscularly and challenged with 800 CFU of *M. marinum* 535. pcDNA and E6C10 served as negative and positive controls, respectively. Statistical analysis: log-rank test. * *p* < 0.05; ** *p* < 0.01; *** *p* < 0.001. (**D**) Bacterial burden (CFU) in zebrafish at 4 weeks post-infection (n = 4). One-way ANOVA. (**E**,**F**) Representative H&E (**E**) and acid-fast staining (AFS, (**F**)) of zebrafish tissues at 4 weeks post-infection. Arrows indicate granulomas (**E**) and mycobacteria (**F**). (**G**) Quantification of granulomas per fish (n = 5). * *p* < 0.05; ** *p* < 0.01; *** *p* < 0.001.

To evaluate the protective efficacy of the engineered recombinant immunogens, we performed in vivo screening using a zebrafish (*Danio rerio*) model of *M. marinum* infection, a widely used surrogate for *M. tuberculosis* [27]. Zebrafish were immunized twice at a 2-week interval with each of the 52 DNA vaccine constructs, a positive control plasmid (pcDNA-E6C10 encoding ESAT-6 and CFP-10), or an empty pcDNA vector. Two weeks following the final immunization, fish were challenged with *M. marinum* strain 535, and survival was monitored for 60 days post-infection (Figure 2A). Kaplan–Meier analysis revealed that 10 of the immunogens, including RI-07, RI-08, RI-10, RI-13, RI-18, RI-19, RI-20, RI-27, RI-31, and RI-36, significantly improved survival relative to the positive control (Figure S2).

To validate these findings, we selected RI-13, RI-20, and RI-31 for independent testing alongside an additional control group co-immunized with pcDNA-Ag85A and pcDNA-E6C10. Western blotting confirmed successful expression of RI-13, RI-20, and RI-31 in HEK293T cells (Figure 2B). Zebrafish received 6 μg DNA vaccine per fish at two-week intervals, followed by challenge with 800 CFU of *M. marinum* 14 days after the final dose. All three immunogens conferred significantly enhanced survival compared to both negative (pcDNA) and positive control groups (E6C10, or Ag85A+E6C10) (Figure 2C). Moreover, at 4 weeks post-infection, bacterial burden analysis in whole-fish homogenates revealed markedly reduced CFUs in RI-13, RI-20, and RI-31 vaccinated groups, indicating effective suppression of bacterial replication (Figure 2D).

Granuloma formation, a hallmark of TB pathology and immune containment, was evaluated through histological analysis at 4 weeks post-infection [28,29]. Granulomas were primarily localized to kidneys, liver, and spleen (Figure 2E,F). All immunized groups showed a significant reduction in granuloma number compared to the negative control (*p* < 0.01), with better outcomes than the E6C10 group (*p* < 0.05) (Figure 2G). Notably, acid-fast staining revealed dense, clustered granulomas in control fish, indicative of uncontrolled bacterial expansion and dissemination. In contrast, granulomas from RI-13, RI-20, and RI-31 immunized fish appeared more discrete, with reduced bacterial load and limited spread, suggesting a shift toward a contained, possibly resolving immune response.

Collectively, these results demonstrate that RI-13, RI-20, and RI-31 confer superior protection against mycobacterial infection in zebrafish, surpassing the widely used ESAT-6/CFP-10 combination and comparable to the Ag85A+E6C10 cocktail, the key immunogen in phase III vaccine GamTBvac [30] (Figure 2C). These findings underscore their potential as promising subunit vaccine candidates targeting both infection control and granuloma modulation.

### 4.3. Recombinant Immunogens RI-13, RI-20, and RI-31 Confer Protection Against M. tuberculosis H37Rv in a Mouse Aerosol Infection Model

**Figure 3:**
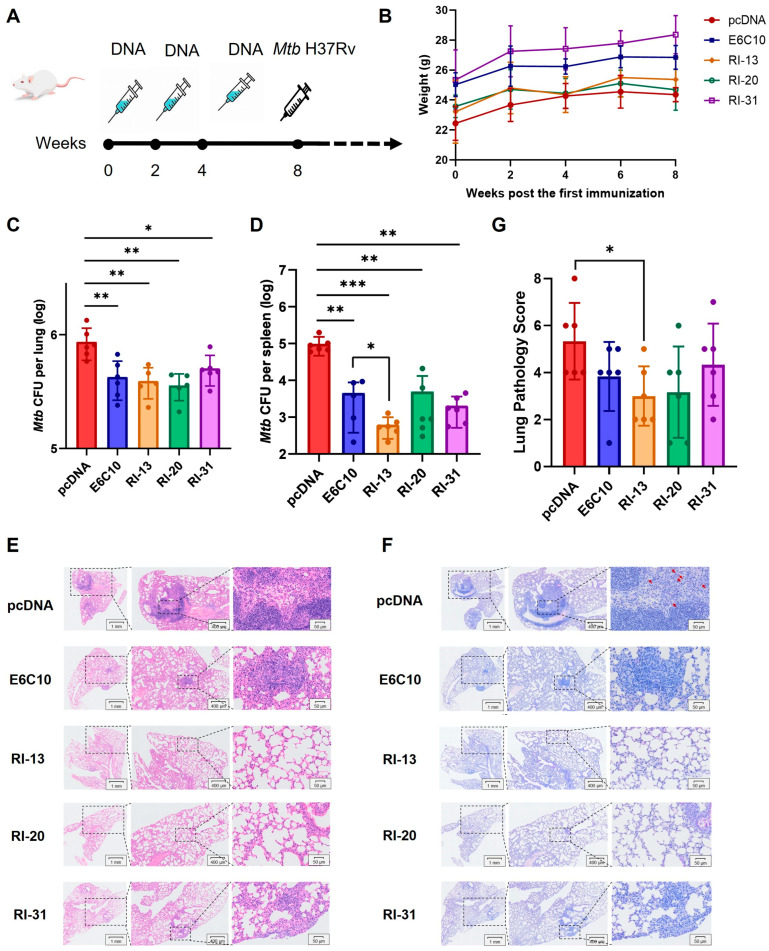
RI-13, RI-20, and RI-31 Protect BALB/c Mice Against *M. tuberculosis* H37Rv Infection. (**A**) Immunization and aerosol infection timeline. (**B**) Body weight monitoring post-immunization. (**C**,**D**) Bacterial burden in lungs (**C**) and spleens (**D**) 4 weeks post-infection (n = 6). pcDNA and E6C10 served as negative and positive controls, respectively. One-way ANOVA. (**E**,**F**) Representative H&E (**E**) and AFS (**F**) staining of lung tissues. Arrows indicate granulomatous lesions and mycobacteria. (**G**) Lung pathology scores based on histological assessment (n = 6). * *p* < 0.05; ** *p* < 0.01; *** *p* < 0.001.

Next, we assessed protective efficacy of the top-performing immunogens in BALB/c mice and profiled vaccine-induced humoral and T helper polarization in C57BL/6 mice to enable mechanistic dissection and to test robustness across genetic backgrounds [31]. BALB/c mice were immunized three times intramuscularly (100 μg/dose, 2-week intervals) with RI-13, RI-20, RI-31, or control plasmids. Four weeks post-final immunization, mice were challenged with *M. tuberculosis* H37Rv via aerosol, and bacterial burden and tissue pathology were assessed 4 weeks later (Figure 3A). Body weight remained stable throughout the experiment, indicating no overt toxicity (Figure 3B).

CFU enumeration revealed that all three recombinant immunogens significantly reduced pulmonary Mtb loads relative to the pcDNA control, with an average reduction of ~0.3 log_10_, comparable to the E6C10 positive control (Figure 3C). Notably, splenic bacterial loads were more substantially reduced. RI-13, RI-20, and RI-31 decreased splenic CFUs by 2.199, 1.027, and 1.679 log_10_, respectively, with RI-13 showing significantly greater efficacy than E6C10 (*p* = 0.0454) (Figure 3D). These findings indicate that all three immunogens, particularly RI-13, mediate systemic protection against disseminated Mtb infection.

Histopathological analysis of H&E-stained lung sections revealed severe alveolar destruction, inflammatory infiltration, and interstitial fibrosis in pcDNA-immunized mice. In contrast, RI-13, RI-20, and RI-31 groups exhibited largely preserved alveolar architecture, with minimal inflammatory lesions predominantly localized to peripheral lung zones (Figure 3E). Acid-fast staining confirmed a marked reduction in Mtb bacilli in immunized groups, consistent with CFU results (Figure 3F). Semi-quantitative histopathological scoring (double-blinded) showed significant amelioration of lung pathology in all immunized groups, with RI-13 achieving the lowest score (3.0 vs. 5.33 in pcDNA, *p* = 0.0199) and the smallest affected alveolar area (43.8%) (Figure 3G). Overall severity ranking from greatest to least was: pcDNA > RI-31 > E6C10 > RI-20 > RI-13. Spleen histology showed intact white pulp, defined follicular structures, and preserved trabeculae across all groups. However, disruption of red/white pulp boundaries and macrophage infiltration was observed in some mice from the pcDNA, RI-20, and RI-31 groups, but not in RI-13 or E6C10 groups, consistent with the spleen bacterial burden data (Figure S3).

In summary, RI-13, RI-20, and RI-31 effectively reduced bacterial burden and mitigated tissue pathology in *M. tuberculosis*-infected mice, with RI-13 demonstrating the most potent protective efficacy across lung and spleen readouts.

### 4.4. RI-13, RI-20, and RI-31 Subunit Vaccines Induce Potent Humoral Immune Responses in Mice

**Figure 4:**
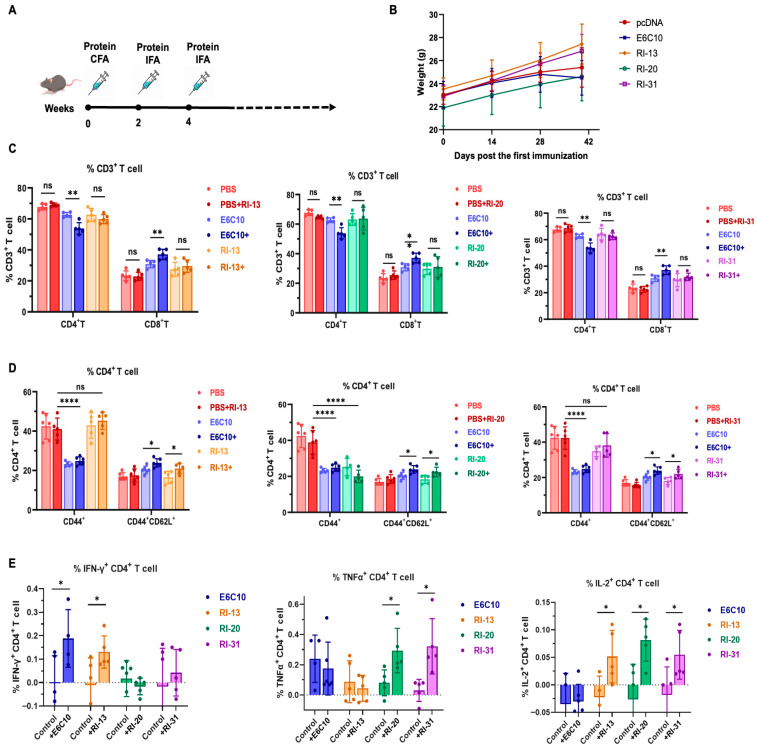
RI-13, RI-20, and RI-31 Subunit Vaccines Induce Humoral and T-Cell Immune Responses in Mice. (**A**) Immunization schedule using protein plus adjuvant. CFA, Complete Freund’s Adjuvant; IFA, Incomplete Freund’s Adjuvant. (**B**) Body weight monitoring post-immunization. (**C**) Frequencies of CD4^+^ and CD8^+^ T cells among CD3^+^ splenocytes (n = 5). “+” indicates ex vivo stimulation with the corresponding antigen. (**D**) Proportions of CD44^+^ and CD44^+^CD62L^+^ (central memory) T cells within CD4^+^ T cells. (**E**) Frequencies of IFN-γ, TNF-α, and IL-2 production by CD4^+^ T cells following antigen stimulation. Unpaired two-tailed *t*-tests were used for statistical analysis. * *p* < 0.05; ** *p* < 0.01; **** *p* < 0.0001.

**Figure 5:**
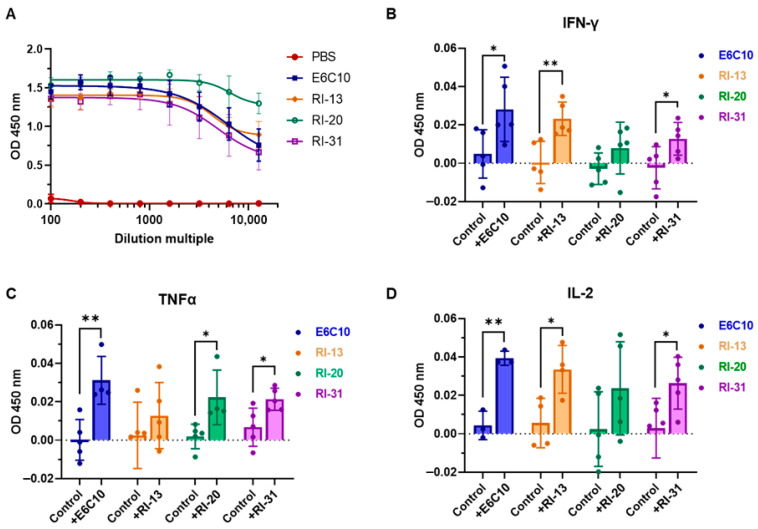
ELISA-based antibody and cytokine analyses in immunized mice. (**A**) ELISA fitting curves showing serum antibody titers from immunized mice, measured across a serial dilution range (1:100 to 1:12,800; n = 6). The PBS group was included as a negative control, and the E6C10 group served as a positive control. (**B**–**D**) ELISA quantification of cytokine secretion by splenic lymphocytes harvested from C57BL/6 mice (n = 5 per group) following immunization with recombinant subunit/adjuvant vaccine formulations. Baseline values from the PBS group were subtracted from each experimental group. E6C10 was included as a positive control. Statistical significance was determined using a two-tailed unpaired *t*-test. * *p* < 0.05; ** *p* < 0.01.

To investigate the immunogenicity of recombinant immunogens in a protein-based vaccine format, we evaluated RI-13, RI-20, and RI-31 as subunit vaccines in C57BL/6 mice. Proteins were expressed and purified to high homogeneity: RI-13, RI-20, RI-31, and E6C10 were produced in HEK293F cells and purified via Ni^2+^ affinity and size-exclusion chromatography (Figure S4A–D). Mice received three intramuscular immunizations (10 µg/dose) at 2-week intervals using protein with complete Freund’s adjuvant for the first dose and incomplete Freund’s for subsequent boosts (Figure 4A). Body weight remained stable across all groups, indicating no adverse systemic effects (Figure 4B). Serum antibody titers were measured by ELISA 12 days after the second and third immunizations. The E6C10 and RI-31 groups showed declining fluorescence signals at a serum dilution of 1:800, while the RI-13 group retained signal up to 1:1600, and the RI-20 group maintained high fluorescence intensity up to 1:12,800 (Figure 5A). These findings indicate that RI-13 and RI-20 elicited stronger antibody responses than E6C10, corresponding to approximately 2-fold and 4-fold increases, respectively.

Collectively, these results demonstrate that RI-13, RI-20, and RI-31 are capable of inducing robust humoral immune responses, with RI-20 exhibiting the highest antibody titers and RI-13 and RI-31 contributing to enhanced immunogenicity relative to the benchmark ESAT-6/CFP-10 antigen.

### 4.5. RI-13, RI-20, and RI-31 Subunit Vaccines Elicit Antigen-Specific T-Cell Responses in Mice

To assess the T-cell-mediated immune responses induced by RI-13, RI-20, and RI-31 subunit vaccines, spleen lymphocytes were harvested from C57BL/6 mice 12 days following the final immunization. Cells were stimulated ex vivo with the corresponding recombinant antigen (10 μL of 0.5 mg/mL), and changes in T-cell subsets and cytokine profiles were analyzed via surface marker and intracellular cytokine staining. Flow cytometric analysis showed that CD4^+^ and CD8^+^ T-cell frequencies within CD3^+^ splenocytes were unchanged across RI-13, RI-20, and RI-31 groups compared to PBS controls. In contrast, stimulation with E6C10 led to a significant decrease in CD4^+^ T cells (*p* = 0.0013) and a concomitant increase in CD8^+^ T cells (*p* = 0.0091), suggesting a more cytotoxic-skewed immune response (Figure 4C). We next evaluated memory T-cell subsets. In RI-13 and RI-31 groups, the proportion of CD44^+^ cells among CD4^+^ T cells remained stable (~34–46%) under both unstimulated and stimulated conditions, whereas both E6C10 and RI-20 groups exhibited reduced CD44^+^CD4^+^ T-cell frequencies (<25%, *p* < 0.0001), suggestive of impaired memory T-cell maintenance. Importantly, stimulation induced a significant increase in central memory T cells (CD4^+^CD44^+^CD62L^+^) in all four vaccinated groups (*p* < 0.05), although E6C10 and RI-20 showed concurrent reductions in effector memory T cells (CD4^+^CD44^+^CD62L^−^), indicating a possible shift away from effector responses (Figure 4D). In contrast, RI-13 and RI-31 preserved effector T-cell populations while expanding central memory T cells, a balance that supports long-term immune recall and sustained vaccine efficacy.

Intracellular cytokine staining further demonstrated that RI-13 significantly elevated IFN-γ (*p* = 0.0471) and IL-2 (*p* = 0.0409) levels in CD4^+^ T cells. RI-20 and RI-31 induced significant increases in TNF-α (*p* = 0.0231 and *p* = 0.0107, respectively), and all three immunogens enhanced IL-2 production to varying extents (Figure 4E). Consistent with these findings, ELISA analysis of cytokines secreted into culture supernatants after 48 h revealed that RI-13 and RI-31 significantly increased IFN-γ (*p* = 0.0067 and *p* = 0.0415), whereas RI-20 had no significant effect. TNF-α levels were elevated following stimulation with RI-20 and RI-31 (*p* = 0.0231 and *p* = 0.0223), and IL-2 secretion was increased in all groups except RI-20 (Figure 5A–D).

In summary, all three subunit vaccines, RI-13, RI-20, and RI-31, elicited antigen-specific Th1-type responses, characterized by increased production of IFN-γ, TNF-α, and IL-2. RI-13 and RI-31, in particular, promoted robust central memory T-cell formation while maintaining effector T-cell homeostasis, indicating their capacity to establish long-lasting, balanced immune memory. RI-13 induced the strongest IFN-γ and IL-2 responses, while RI-20 and RI-31 favored TNF-α-dominated responses.

## 5. Discussion

Tuberculosis remains a major global health challenge, compounded by the limited protection of BCG against adult pulmonary disease and the growing burden of drug-resistant *Mycobacterium tuberculosis* (Mtb) [12,32]. In this study, we established a bioinformatics-guided, multi-epitope immunogen engineering and screening framework and evaluated three recombinant candidates, RI-13, RI-20, and RI-31. Across zebrafish and murine models, these immunogens conferred measurable protection and reduced disease-associated pathology, supporting their potential as next-generation TB vaccine leads (Figure 1, Figure 2, Figure 3, Figure 4 and Figure 5).

A key strength of the work is the end-to-end prioritization strategy that couples computational antigen discovery to rapid in vivo triage. Using combined B-cell and T-cell epitope prediction and chimeric immunogen design, we identified 74 candidate antigens from the annotated Mtb proteome. In the zebrafish infection model, 10 engineered constructs outperformed the positive control E6C10 (Figure 2), highlighting the utility of zebrafish as a scalable, intermediate throughput platform to de-risk candidate selection before resource-intensive mammalian studies. Due to P3 facility constraints, we advanced three candidates for deeper immunogenicity and protection analyses, although additional constructs among the remaining seven may also warrant follow up (Figure 3, Figure 4 and Figure 5).

The three selected immunogens incorporate distinct antigen modules on an E6C10 backbone (Table S1): RI-13 includes epitopes derived from the lipoprotein LppO, RI-20 includes epitopes from the ESAT-like protein EsxN, and RI-31 includes epitopes from the low molecular weight T cell antigen TB8.4. These antigens have been reported to elicit immune responses in different infection contexts [11,33,34,35,36,37], and their combination with canonical ESAT-6 and CFP-10 derived components may broaden epitope coverage and increase the probability of capturing protective T cell specificities in treating TB.

Mechanistically, our data suggest that protection is associated with coordinated humoral and cellular responses. RI-20 induces the highest antibody titers, yet this does not indicate superior immune protection. Instead, the subtype distribution of elicited IgG antibodies should also be taken into account. RI-13 showed the most consistent immunogenicity profile, with increased IFN-γ and IL-2, cytokines linked to macrophage activation and antibacterial effector functions (Figure 3, Figure 4 and Figure 5). In addition, RI-13 and RI-31 promoted a central memory T cell compartment while maintaining effector populations, a pattern often associated with durable protection. Together, these observations support the concept that rationally assembled multi-epitope immunogens can approximate aspects of natural antigenic complexity while enabling controlled composition, potentially improving the breadth and quality of vaccine-induced immunity [38,39].

Several limitations should be considered when interpreting these findings. First, the zebrafish-*Mycobacterium marinum* infection model was adopted for initial high-throughput screening in this study. This model is a powerful tool for investigating mycobacterial pathogenesis and host immune responses, with prominent advantages in rapid screening during infection. Nevertheless, there are considerable critical differences between this model and human tuberculosis. For instance, zebrafish possess a unique immune system, and their adaptive immune components, T-cell subsets, and cytokine profiles differ markedly from those of humans. Significant discrepancies also exist in granuloma formation, bacterial control mechanisms, and the capacity to mimic human latent tuberculosis infection. Accordingly, the zebrafish model in our study was merely utilized for high-throughput antigen preliminary screening. Second, in the H37Rv challenge model, the lung CFU reduction in mice was approximately 0.3 log_10_, which is not a particularly striking result. However, we found that RI-13 exhibited prominent efficacy in reducing splenic bacterial burden in mice. The spleen is the central organ for B-cell immunity, which verifies our original rationale for antigen design: we aimed to focus not only on T-cell immunity but also on anti-tuberculosis effects mediated by B-cell immunity. In this study, we intended to induce the production of circulating antibodies by incorporating screened B-cell epitopes, thereby enhancing overall anti-tuberculosis immune protection. In addition, our murine experiments used a DNA vaccine delivery format. The protective efficacy of protein plus adjuvant formulations or BCG booster vaccine, which are more aligned with common clinical development paths, remains to be evaluated in challenge settings [40]. Second, mechanistic resolution could be strengthened by defining vaccine-responsive cell states and antigen-specific clonal expansion using single-cell transcriptomics and paired TCR sequencing, and by mapping key epitopes to MHC binding and presentation using structural or biochemical approaches [41].

Despite these limitations, this study provides a practical and scalable pipeline for TB vaccine discovery that integrates computational epitope prioritization, in vivo screening in zebrafish, validation in mice, and mechanistic immune profiling (Figure 1). By reducing candidate attrition earlier and focusing downstream resources on the most promising constructs, the framework may accelerate iterative optimization. RI-13 emerges as a lead candidate within the set, and future work should prioritize head-to-head comparison of delivery formats, durability, and protection in aerosol challenge models, alongside systems-level immune profiling to identify correlates of protection.

## 6. Conclusions

This study establishes an artificial intelligence-guided strategy for the rational discovery and prioritization of novel *Mycobacterium tuberculosis* vaccine immunogens. By integrating epitope prediction with stepwise in vivo validation, we identified RI-13, RI-20, and RI-31 as leading candidates with clear protective efficacy and strong immunogenicity across zebrafish and mouse models. Among them, RI-13 emerged as the most promising candidate, showing superior overall immune activation and a balanced Th1, Th2, and Th17 response profile, together with measurable reductions in bacterial burden and tissue pathology. These findings not only support RI-13 as a next-generation TB vaccine candidate for further development but also demonstrate the broader value of AI-guided immunogen design as a scalable framework for accelerating vaccine discovery and preclinical selection against TB.

## Data Availability

The original contributions presented in this study are included in the article/Supplementary Materials. Further inquiries can be directed to the corresponding authors.

## Supplementary Materials

The following supporting information can be downloaded at: https://www.mdpi.com/article/10.3390/vaccines14050408/s1, Figure S1. Strategy for Constructing Two Anti-Tuberculosis Recombinant Immunogens; Figure S2. Survival curves of adult zebrafish post infection; Figure S3. Representative hematoxylin and eosin (H&E) staining of spleen sections from mice at 4 weeks following infection with *Mycobacterium tuberculosis* H37Rv; Figure S4. Size-exclusion chromatography profiles and SDS-PAGE analyses of the purified recombinant proteins; Table S1. Gene IDs and function description of selected 74 antigens of Mtb; Table S2. 52 recombinant immunogens expressed in eukaryotic cells and their corresponding ID numbers.
