# Supplementary material for: Recombinant Immunogens Designed by AI Epitope Prioritization Confer Protection Against Mycobacterium tuberculosis
Source: Vaccines (Basel). 2026 May 1;14(5):408. doi: 10.3390/vaccines14050408 (PMC13211660; doi:10.3390/vaccines14050408)
Supplement: Supplementary file 1 [file vaccines-14-00408-s001.zip › Supplementary Materials.pdf]

Supplementary Materials

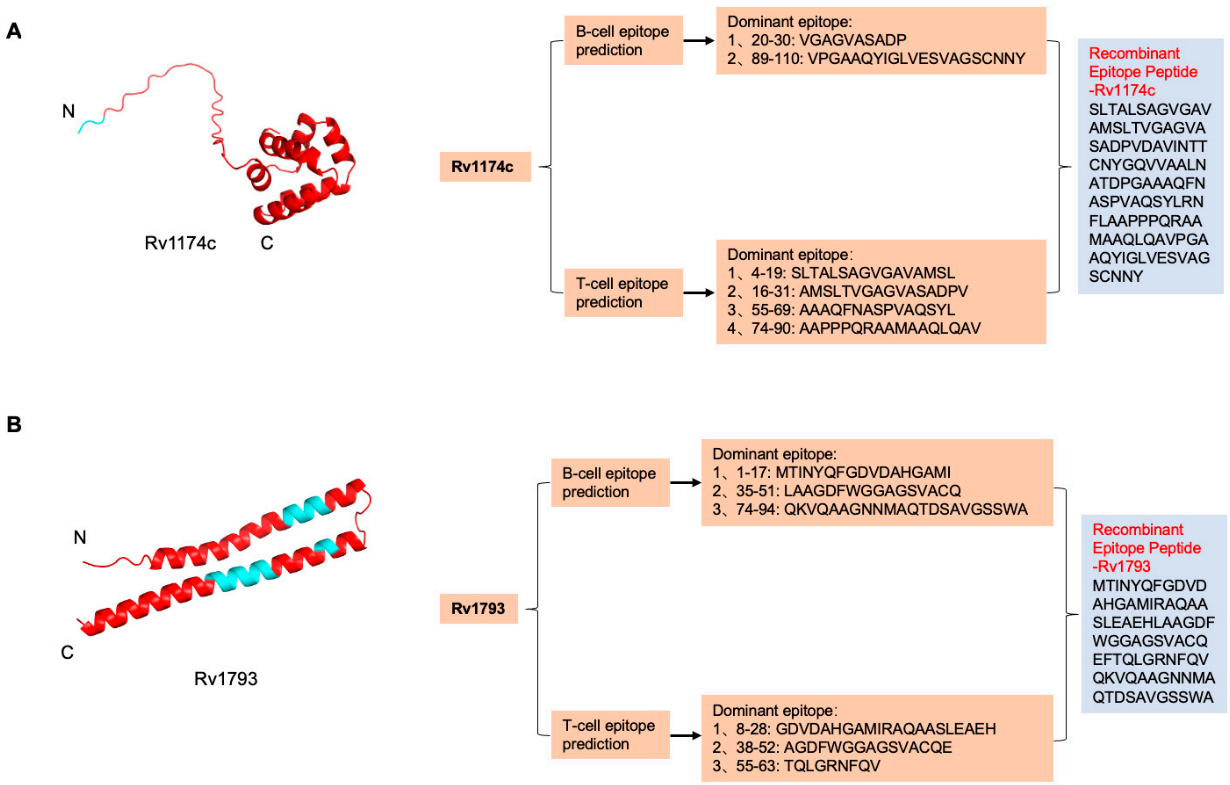

Figure S1. Strategy for Constructing Two Anti-Tuberculosis Recombinant Immunogens.

(**A**) Structural prediction of Mtb Rv1174c using AlphaFold3. Predicted epitope regions are highlighted in red. Human T-cell and B-cell epitopes were identified using the IEDB database and SEPPA 3.0, respectively. The blue panel to the right illustrates the rational recombination of predicted Rv1174c epitopes to design candidate recombinant vaccine antigens. (**B**) Structural prediction of Mtb Rv1793 using AlphaFold3, with predicted epitope regions highlighted in red. Human T-cell and B-cell epitopes were similarly predicted using IEDB and SEPPA 3.0. The blue panel on the right shows the rational recombination of predicted Rv1793 epitopes for candidate vaccine antigen design.

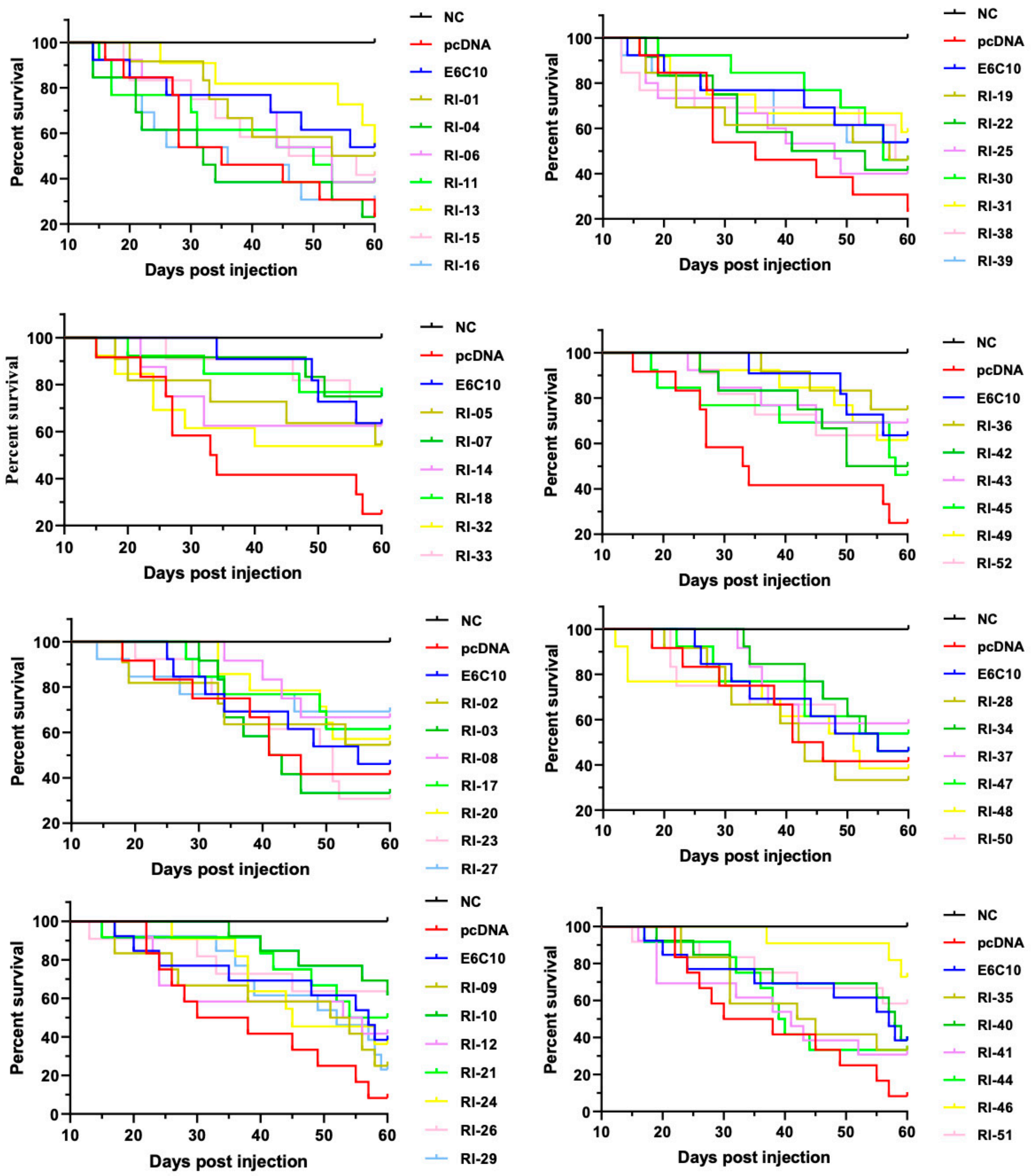

Figure S2. Survival curves of adult zebrafish post infection. Adult zebrafish (n = 15 per group) immunized intramuscularly with each of 54 recombinant immunogens twice at a 2-week interval, followed by intraperitoneal challenge with 800 CFU of *Mycobacterium marinum* 535. Zebrafish injected with pcDNA served as negative controls, and E6C10-immunized fish as positive controls.

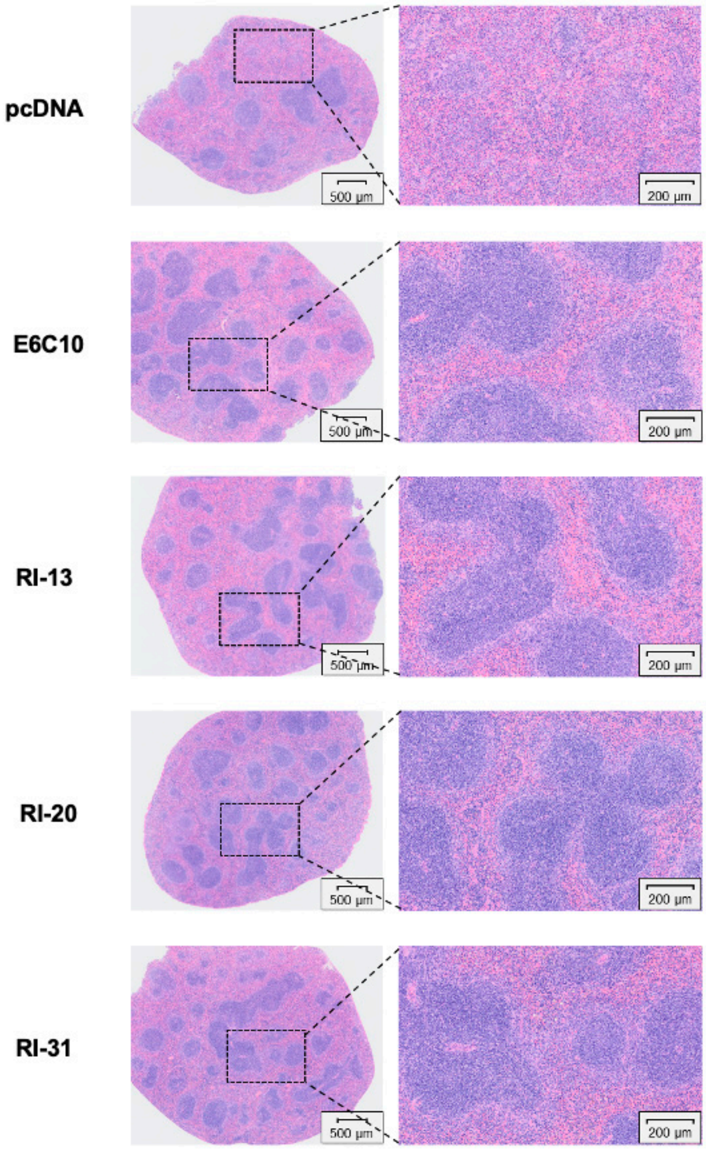

Figure S3. Representative hematoxylin and eosin (H&E) staining of spleen sections from mice at 4 weeks following infection with *Mycobacterium tuberculosis* H37Rv.

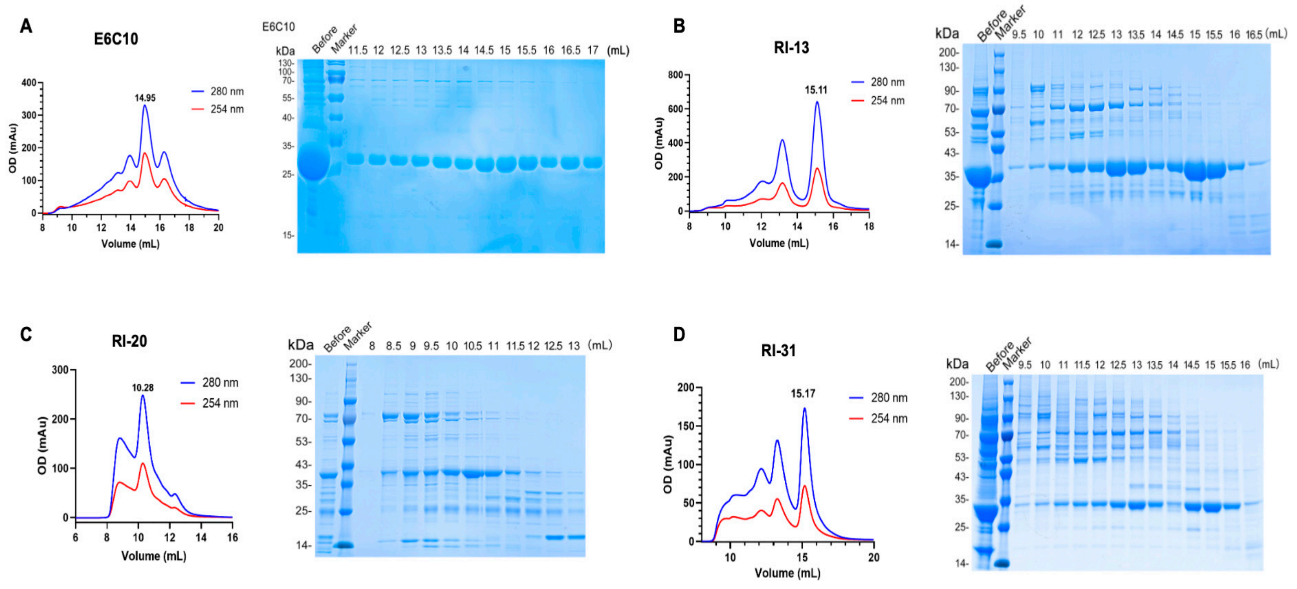

Figure S4. Size-exclusion chromatography profiles and SDS-PAGE analyses of the purified recombinant proteins.

(**A**) E6C10, (**B**) RI-13, (**C**) RI-20, and (**D**) RI-31 were purified using a Superdex 200 10/300 column, with elution fractions subsequently analyzed by SDS-PAGE to confirm purity and integrity.

Table S1. Gene IDs and function description of selected 74 antigens of Mtb.

Gene	Protein and Function
Rv0125	Probable serine protease PepA
Rv0129c	mycolyltransferase Ag85C
Rv0200	Possible conserved transmembrane protein
Rv0203	Heme-binding protein Rv0203
Rv0222	Probable enoyl-CoA hydratase EchA1
Rv0287	ESAT-6-like protein EsxG
Rv0288	ESAT-6-like protein EsxH
Rv0309	Possible conserved exported protein
Rv0440	Chaperonin GroEL 2
Rv0667	DNA-directed RNA polymerase subunit beta
Rv0670	Probable endonuclease IV
Rv0934	Phosphate-binding protein PstS 1
Rv1157c	Conserved ala-, pro-rich protein
Rv1158c	Conserved hypothetical ala-, pro-rich protein
Rv1174c	Low molecular weight T-cell antigen TB8.4
Rv1184c	Diacyltrehalose acyltransferase Chp2
Rv1198	ESAT-6-like protein EsxL
Rv1242	Ribonuclease VapC33
Rv1255c	Uncharacterized HTH-type transcriptional regulator Rv1255c
Rv1256c	Cytochrome P450 130
Rv1280c	Uncharacterized protein Rv1280c
Rv1291c	Uncharacterized protein Rv1291c
Rv1623c	Probable integral membrane cytochrome D ubiquinol oxidase (Subunit I) CydA
Rv1641	Translation initiation factor IF-3
Rv1694	16S/23S rRNA (cytidine-2'-O)-methyltransferase TlyA
Rv1793	ESAT-6-like protein EsxN
Rv1860	Alanine and proline-rich secreted protein Apa
Rv1886c	Diacylglycerol acyltransferase Ag85B
Rv1926c	Immunogenic protein MPT63
Rv1945	Uncharacterized protein Rv1945
Rv1980c	Immunogenic protein MPT64
Rv1985c	HTH-type transcriptional regulator LysG
Rv1986	Lysine exporter LysE
Rv1987	Uncharacterized protein Rv1987
Rv2031c	Alpha-crystallin hspX
Rv2182c	1-acylglycerol-3-phosphate O-acyltransferase
Rv2190c	Probable endopeptidase Rv2190c
Rv2220	Glutamine synthetase glnA1
Rv2223c	Carboxylesterase B
Rv2290	Putative lipoprotein LppO
Rv2346c	ESAT-6-like protein EsxO
Rv2351c	Phospholipase C A
Rv2476c	NAD-specific glutamate dehydrogenase
Rv2654c	Antitoxin Rv2654c
Rv2658c	Uncharacterized protein Rv2658c
Rv2666	Mutator family transposase
Rv2715	Uncharacterized protein Rv2715
Rv2780	Alanine dehydrogenase
Rv2823c	CRISPR system single-strand-specific deoxyribonuclease Cas10/Csm1 (subtype III-A)
Rv2875	Immunogenic protein MPT70
Rv2878c	Soluble secreted antigen MPT53
Rv2903c	Phosphoprotein
Rv2945c	Putative phthiocerol dimycocerosate transporter LppX
Rv3017c	ESAT-6-like protein EsxQ
Rv3019c	ESAT-6-like protein EsxR
Rv3106	NADPH-ferredoxin reductase FprA
Rv3296	Probable ATP-dependent helicase Lhr
Rv3333c	Hypothetical proline rich protein
Rv3378c	Tuberculosinyl adenosine transferase
Rv3418c	Co-chaperonin GroES
Rv3467	HNH nuclease domain-containing protein
Rv3497c	Carboxylesterase/phospholipase LipF
Rv3619c	ESAT-6-like protein EsxV
Rv3689	Probable conserved transmembrane protein
Rv3714c	Cullin, a subunit of E3 ubiquitin ligase
Rv3803c	MPT51/MPB51 antigen
Rv3804c	Diacylglycerol acyltransferase Ag85A
Rv3812	PE-PGRS family protein PE_PGRS62
Rv3846	Superoxide dismutase [Fe]
Rv3871	ESX-1 secretion system protein EccCb1
Rv3873	PPE family immunomodulator PPE68
Rv3874	10 kDa culture filtrate antigen CFP-10
Rv3875	6 kDa early secretory antigenic target ESAT-6
Rv3879c	ESX-1 secretion-associated protein EspK

Table S2. 52 recombinant immunogens expressed in eukaryotic cells and their corresponding ID numbers.

Gene	Recombinant Immunogen ID
Rv0125	RI-01
Rv0129c	RI-02
Rv0200	RI-03
Rv0222	RI-04
Rv0287	RI-05
Rv0288	RI-06
Rv0309	RI-07
Rv0667	RI-08
Rv0670	RI-09
Rv0934	RI-10
Rv1157c	RI-11
Rv1158c	RI-12
Rv1174c	RI-13
Rv1184c	RI-14
Rv1198	RI-15
Rv1255c	RI-16
Rv1291c	RI-17
Rv1641	RI-18
Rv1694	RI-19
Rv1793	RI-20
Rv1886c	RI-21
Rv1926c	RI-22
Rv1945	RI-23
Rv1985c	RI-24
Rv1986	RI-25
Rv2031c	RI-26

Gene	Recombinant Immunogen ID
Rv2182c	RI-27
Rv2190c	RI-28
Rv2220	RI-29
Rv2223c	RI-30
Rv2290	RI-31
Rv2346c	RI-32
Rv2351c	RI-33
Rv2476c	RI-34
Rv2658c	RI-35
Rv2666	RI-36
Rv2715	RI-37
Rv2823c	RI-38
Rv2875	RI-39
Rv2878c	RI-40
Rv2903c	RI-41
Rv2945c	RI-42
Rv3017c	RI-43
Rv3296	RI-44
Rv3378c	RI-45
Rv3418c	RI-46
Rv3467	RI-47
Rv3619c	RI-48
Rv3714c	RI-49
Rv3803c	RI-50
Rv3873	RI-51
Rv3879c	RI-52
